# ROS-Activated homodimeric podophyllotoxin nanomedicine with self-accelerating drug release for efficient cancer eradication

**DOI:** 10.1080/10717544.2021.1995076

**Published:** 2021-11-08

**Authors:** Bingfeng Liang, Dangxia Zhou

**Affiliations:** a Department of Pathology, School of Basic Medicine, Xi’an Jiaotong University Health Science Center, Xi’an, China; b Department of Nursing, Hebei Women's Vocational College, Shijiazhuang, China

**Keywords:** Dimeric prodrug, ROS generation, vitamin K3, high drug loading, tumor-specific drug release

## Abstract

Although podophyllotoxin (POD) demonstrates high efficiency to inhibit various cancers, its clinic application is limited to poor bioavailability. Nanoparticles derived from homodimeric prodrugs with high drug loading potential are emerging as promising nanomedicines. However, complete intracellular drug release remains a major hindrance to the use of homodimeric prodrugs-based nanomedicine. We sought to develop a reactive oxygen species (ROS) responsive POD dimeric prodrug by incorporating vitamin K3 (VK3) and Pluronic F127 to synthesize a spheroid nanoparticle (PTV-NPs). PTV-NPs with high POD content could release drugs under the ROS enrichment microenvironment in cancer cells. The released VK3 could produce abundant ROS selectively in tumor cells catalyzed by the overexpressed NAD(P)H: quinone oxidoreductase-1 (NQO1) enzyme. In turn, the resultant high ROS concentration promoted the conversion of POD dimeric prodrug to POD monomer, thereby achieving the selective killing of cancer cells with weak system toxicity. *In vitro* and *in vivo* studies consistently confirmed that PTV-NPs exhibit high drug loading potential and upstanding bioavailability. They are also effectively internalized by tumor cells, induce abundant intracellular ROS generation, and have high tumor-specific cytotoxicity. This ROS-responsive dimeric prodrug nanoplatform characterized by selective self-amplification drug release may hold promise in the field of antitumor drug delivery.

## 1. Introduction

Cancer is a major threat to human health. Chemotherapy is one of the primary treatment modalities available for cancer patients. However, the present status of chemotherapy is far from being satisfactory, which is seriously limited to the poor water solubility and the side effects (Zuo et al., 2020). Podophyllotoxin (POD), a natural aryltetralin lignan, is an extract of Podophyllum hexandrum, which has been demonstrated to be effective against cancer (Feng et al., 2020; Zhao et al., 2021). POD can specifically bind to tubulin in the cell division process, inhibiting the formation of mitotic spindles (Zhang et al., 2018; Kumbhar et al., 2020). There is evidence that poor water solubility and highly off-target toxicity hinder POD application in clinics (Ou et al., 2019; Liu et al., 2020). Hence, it is imperative to develop a simple delivery system to decrease POD toxicity and improve its antitumor effects efficiently.

Currently, a nanotechnology-based drug delivery system (NDDS) has seen unprecedented use in the cancer field to improve bioavailability and reduce the side effect of anticancer drugs (Ding et al., 2017). Several NDDSs have been developed and used in the clinics, including Genexol-PM (paclitaxel-loaded polymeric micelle) and Doxil (pegylated-liposomal doxorubicin) (Xu et al., 2017; Hossen et al., 2020). Conventional NDDS prepared through noncovalent encapsulation of drugs in nanocarriers is characterized by carrier-related toxicities, low drug loading efficiency, and premature drug release during circulation (Tang et al., 2021). Additionally, the complicated preparation techniques are a huge hindrance to the application of NDDS in clinical practice (Duncan & Vicent, 2010). Thus, developing an efficient and straightforward NDDS is of promise to address these challenges.

Recent evidence shows that NDDS developed using prodrugs has gained much attention (Huang et al., 2021). These NDDSs are more advantageous due to their facile preparation, good reproducibility, and high drug loading (Zhang et al., 2021). Homodimer prodrugs self-assembled nanomedicines, a new branch of NDDS with a clear chemical structure, have been the focus of research in the field of anticancer drug delivery (Li et al., 2020). Of note, dimeric prodrugs are the inactive conjugations of two drug molecules, which have advantages of higher drug loading capability, in addition to those of prodrug-based NDDS (Sun et al., 2019). Compared with monomer prodrug NDDS, the homodimer-prodrug formed NDDS (HDDNS) exhibit much higher drug loading capacities (Li et al., 2020). Despite the multiple advantages presented by homodimer-prodrug-based NDDS, its clinical use is hampered by several challenges, especially incomplete drug release (Xu et al., 2017; Feng et al., 2020).

Improving the therapeutic efficacy of HNDDS requires an effective strategy of introducing stimuli responsiveness to make them respond to tumor microenvironments (such as reactive oxygen species (ROS), glutathione, enzymes, acidity, hypoxia, etc.) and completely release drugs (Mura et al., 2013; van der Meel et al., 2019). Among these stimuli, ROS has attracted extensive interest because of their higher concentration (up to 10 folds) in cancer cells than normal cells (Ye et al., 2017; Li et al., 2018). Thereby, a homodimer prodrug with ROS-sensitive linkages is a powerful strategy to achieving tumor-specific drug delivery. Recent evidence shows that a mass of ROS-sensitive linkers, including alkylene sulfide, thioketal (TK), and boronic ester, have been used to construct ROS-activable nanomedicines for cancer management (Saravanakumar et al., 2017). However, the heterogeneity of tumor hinder the application of ROS-responsive nanomedicine, because of the endogenous ROS level is too low to trigger drug release (Dai et al., 2019; Yin et al., 2019). Thus, ROS-responsive HNDDS with ROS production capability is an alternative approach that holds promise to significantly enhance drug release selectivity, increase anti-cancer efficacy, and reduce side effects.

Reports show that vitamin K3 (VK3), also called menadione, has superior tumor-activating ROS production ability (Yang et al., 2018). NAD(p)H: quinone oxidoreductase-1 (NQO1) enzyme catalyzes VK3 reduction to semiquinone and hydroquinone, whose oxidation to quinone generates ROS ( Dasari et al., 2017; Xu et al., 2020). Thus, the cyclic conversion among VK3, semiquinone, and hydroquinone present an effective strategy to increase ROS concentration in cells (Yang et al., 2018). Moreover, the expression of NQO1 is 100-folds more in cancer cells than in normal cells (Ma et al., 2015). These data show that VK3 is a promising tumor-specifical ROS generator.

**Scheme 1.**
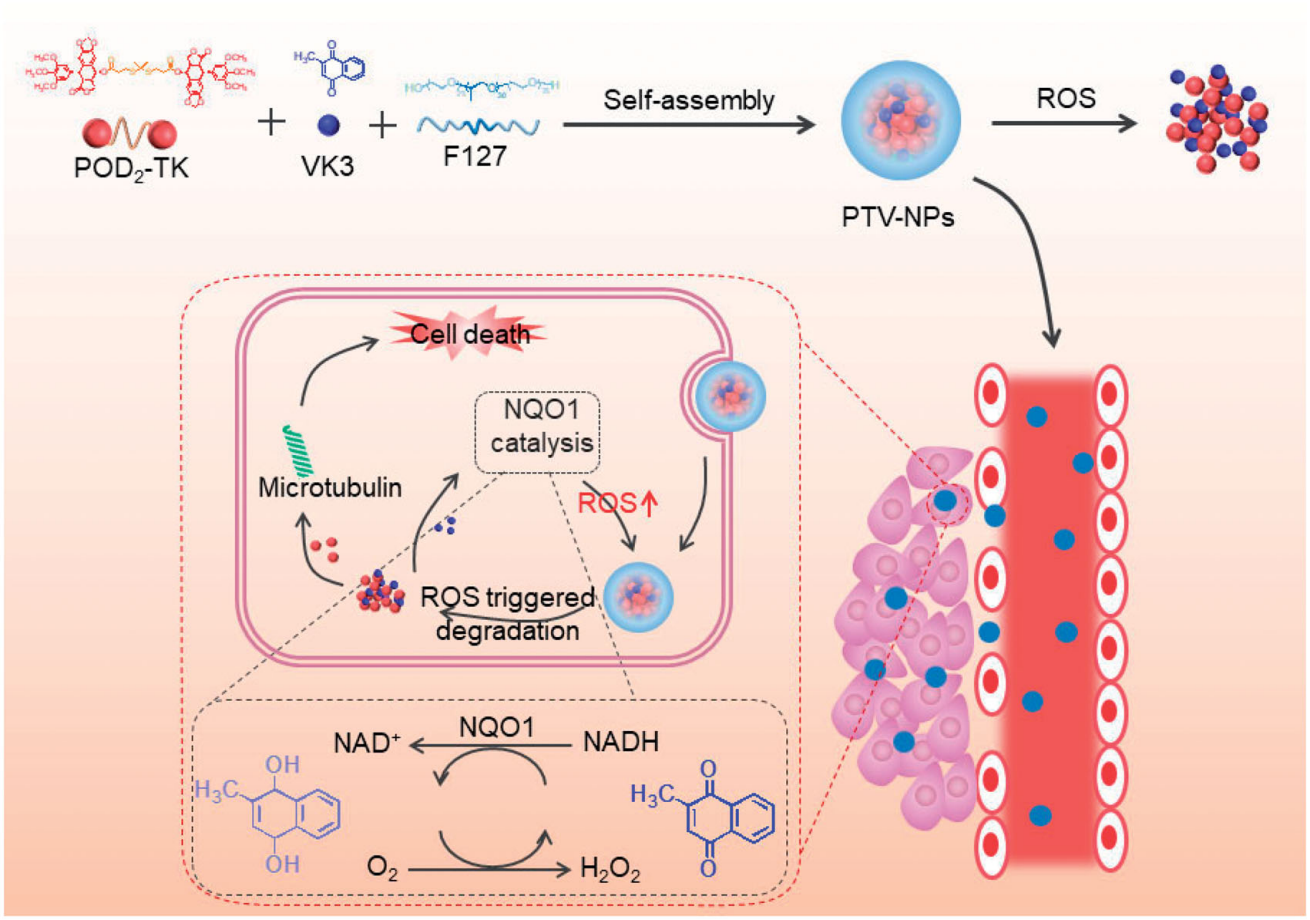
Schematic illustration of PTV-NPs preparation and intracellular self-amplification drug release.

Herein, we develop a ROS-responsive HNDDS (denoted as PTV-NPs) with self-amplifying drug release potential by encapsulating POD homodimer-prodrug and VK3 into a biocompatible polymer (Pluronic F127) formed nanoparticles (NPs) (Scheme 1). After accumulation in the tumor site through the enhanced permeability and retention (EPR) effect, PTV-NPs could release a small amount of POD and VK3 under the triggers of endogenous ROS. Subsequently, the released VK3 generates ROS under the catalysis of NQO1, which in turn amplifies drug release and NPs collapse. These events allow for efficient conversion of POD, which induces cancer cell death. In normal cells, due to low ROS concentration and expression of NQO1, VK3 is not enough to generate sufficient ROS. As such, the POD conversion process is interrupted, resulting in low cytotoxicity of the PTV-NPs against normal cells. These data demonstrate that integrating multiple mechanisms into a single PTV-NPs would overcome the drawbacks of POD, reduce the side effects of POD, and substantially improve therapeutic efficacy.

## 2. Materials and experiments

### 2.1. Materials

Podophyllotoxin (POD), 4-dimethylaminopyridine (DMAP), 1-Ethyl-3-(3-dimethylaminopropyl) carbodiimide hydrochloride (EDC⸱HCl), acetone, 3-mercaptopropionic acid, vitamin K3 (VK3), dicoumarol (DIC), and anhydrous dimethyl sulfoxide (DMSO) were purchased from Aladdin Reagent Co. Ltd. (Shanghai, China). ROS Assay Kit (dichlorofluorescindiacetate, DCFH-DA), Cell Counting Kit-8 (CCK-8), and Hoechst 33342 were purchased from Beyotime Biotechnology Co. Ltd. (Shanghai, China). Pluronic F-127 was purchased from Sigma-Aldrich (USA).

### 2.2. Thioketal (TK) synthesis

TK was prepared as described previously with several modifications (Hu et al., 2017). In brief, anhydrous 3-mercaptopropionic acid (6.0 g) and anhydrous acetone (6.8 g) were stirred in dry hydrogen chloride for 6 h at room temperature. The resultant mixture was crystalized at −30 °C for 48 h. The mixture was filtered, and the obtained solid products were washed with hexane and cold water five times, respectively, and finally dried under vacuum to obtain TK. The molecular structure of TK was measured by proton nuclear magnetic resonance (^1^H NMR, Bruker AV 600 M) was used to confirm the molecular structure of TK. The mass spectrum (MS, QTRAP 5500 triple quadrupole mass spectrometer, AB SCIEX^TM^, USA) was utilized to determine the molecular weight of TK.

### 2.3. POD2-TK synthesis

A condensation reaction between POD and TK allowed for POD_2_-TK synthesis. Briefly, POD (212.0 mg, 0.50 mmol), TK (55.4 mg, 0.22 mmol), EDC⸱HCl (201.7 mg, 1.06 mmol), and DMAP (73.2 mg, 0.6 mmol) were dissolved in anhydrous DMSO. The solution was stirred for 1 h at 30 °C under nitrogen protection. Then, DMAP (36.6 mg, 0.3 mmol) and EDC⸱HCl (99.8 mg, 0.58 mmol) were added, and the mixture was left to react for another 48 h. The reaction product was purified by silica gel column chromatography using ethyl acetate and dichloromethane (2/3, v/v) as eluent. POD_2_-TK (with 71.2% yield) was obtained as a white solid.

The ROS-insensitive alkyl linker bridged POD dimmer, denoted as POD_2_-CC, was prepared by the same method, except TK was substituted by azelaic acid (yield: 67.3%). The molecular structure and weight of POD_2_-TK and POD_2_-CC were verified by ^1^H NMR, ^13 ^C NMR, and MS.

### 2.4. Preparation of NPs

Drug-loaded NPs were produced by the nanoprecipitation method. First, drug solutions were prepared as follows. Solution A: POD_2_-TK or POD_2_-CC in DMSO (10 mg/mL); solution B: VK3 in DMSO (2 mg/mL); solution C: F127 in DMSO (20 mg/mL). Next, solutions A (0.6 mL), B (0.1 mL), and C (0.4 mL) were mixed thoroughly. To the mixture, 10.0 mL of water was added under vigorous stirring. The unencapsulated drugs and DMSO were removed through centrifuge dialysis (Mw: 30 kDa) and filtered through a 220 nm filter. The NPs were coded based on their composition as follows: PTV-NPs (POD_2_-TK, VK3, and F127); PCV-NPs (POD_2_-CC, VK3, and F127); PT-NPs (POD_2_-TK and F127). As a control, the free POD-loaded NPs (denoted as POD-NPs) were also prepared using the same method by adding the mixture of 0.6 mL POD solution (10 mg/mL) and 0.4 mL of F127 solution (20 mg/mL) into 10 mL water. Parameters of NPs, including zeta-potential, size, and size distribution were detected via the dynamic light scattering (DLS) method with Malvern Zeta Nano sizer. The morphology of the NPs was examined by transmission electron microscopy (TEM) using a JEOL JEM-1011 electron microscope. The drug loading content (DLC) and loading efficiency (DLE) of NPs were evaluated by high-performance liquid chromatography (HPLC, Shimadzu, LC-20A) in an analytical column (Agilent ODS C18 column, 4.6 × 250 mm, 2.5 μm particle size). For POD analysis, the mobile phase was water: acetonitrile (43/57, v/v, 0.1% TFA) at a flow rate of 1 mL/min. A UV detector, set at 254 nm, was employed to monitor the column effluent. For VK3 analysis, the mobile phase was water: acetonitrile (30/70, v/v) at a flow rate of 1 mL/min, and UV detection at 265 nm was used.

### 2.5. Evaluating ROS-responsive ability of NPs

The ROS-responsive potential of NPs was investigated by DLS. PTV-NPs and PCV-NPs were incubated at 37 °C in PBS (pH 7.4) or PBS (pH 7.4) containing 10.0 mM H_2_O_2_. After treatment for 12 h, the size of NPs was detected by DLS.

### 2.6. *In vitro* drug leakage

*In vitro* drug release was studied based on a previously described ultrafiltration centrifugation method (Jiang et al., 2017). The release medium comprised PBS (pH 7.4) with 0, 1, or 10 mM H_2_O_2_ supplemented with 0.2% (w/v) Tween-80. Briefly, fresh prepared NPs were dispersed in the release medium (4 mL) at a concentration of 0.2 mg/mL (equal to POD). The sample was maintained at 37 °C with slight shaking. At different time points, the solutions were transferred to centriprep centrifugal filter units (MWCO = 10 kDa), centrifuged at 5,000 rpm for 10 min to obtain free forms. The released drug was quantified by the HPLC method as described in the above section.

### 2.7. *In vitro* stability evaluation

For colloid stability evaluation, drug-loaded NPs were maintained at 37 °C in PBS (pH 7.4) with or without 10% FBS with slight stirring. Changes in particle size and size distribution of NPs were detected at pre-set time points by the DLS method.

### 2.8. Cells and animals

Human breast cancer MCF-7 cells and mouse embryonic fibroblast NIH-3T3 cells were obtained from the National Collection of Authenticated Cell Cultures (Shanghai, China) and cultured in DMEM in a humidified atmosphere containing 5% CO_2_ at 37 °C.

BALB/c nude mice (4-week-old, female) were purchased from Vital River Laboratories (Beijing, China). The Animal Care and Use Committee of Xi’an Jiaotong University approved all study protocols.

### 2.9. Cellular uptake analysis

The cellular uptake process was observed by a confocal laser scanning microscope (CLSM, Zeiss LSM 700, Zurich, Switzerland). Before LCSM analysis, the coumarin-6 loaded PT-NPs, PTV-NPs, and PCV-NPs were prepared. Briefly, 6 mg of the prodrug, 8 mg or F127, and 200 µg coumarin-6 were dissolved in DMSO and mixed thoroughly. Subsequently, the mixture was added to 10.0 mL of water under vigorous stirring. The unencapsulated drugs and DMSO were removed through centrifuge dialysis (Mw: 30 kDa) and filtered through a 220 nm filter. The obtained NPs were centrifugated at 8,000 rpm using an ultrafiltration centrifuge tube (Mw: 10 kDa) and then resuspended in cell culture. For CLSM analysis, cells were seeded in the glass bottom dishes (1 × 10^5^ cells per well). After 24 h culture, we treated the cells with above-prepared coumarin-6 loaded PT-NPs, PTV-NPs, or PCV-NPs for 1 or 4 h, respectively. Subsequently, cells were rinsed with PBS four times, stained by Hoechst 33342 at room temperature for 15 min, fixed with 4% paraformaldehyde at room temperature for 15 min, and examined by CLSM.

### 2.10. Intracellular ROS generation

We assessed intracellular ROS production by CLSM and flow cytometry (Beckman, USA), with dichlorofluorescindiacetate (DCFH-DA) as a probe. Firstly, the potential of VK3-induced ROS production was quantified by flow cytometry. Briefly, cells were seeded onto 6-well plates (1 × 10^5^ cells/well) and cultured for 1 day. The cells were treated with VK3 at different doses (0, 0.5, 1, 2, 3, or 4 μg/mL) with or without DIC for 12 h, or incubated with 2 μg/mL VK3 for various times (0, 2, 4, 8, 12, or 24 h). Subsequently, the cells were incubated with DCFH- DA at 37 °C for 30 min. The cells were then digested by trypsin, collected through centrifugation, and washed with PBS. A flow cytometer was used to analyze the stained cells.

NPs-induced ROS production was investigated by CLSM and flow cytometry. In the CLSM experiment, cells were seeded in the glass bottom dishes (1 × 10^5^ cells/well). After overnight cultivating, cells were treated with POD, PT-NPs, PTV-NPs, or PCV-NPs (equal to 2 μg/mL of VK3) for 12 h, respectively. Then, the cells were treated with DCFH-DA for 30 min at 37 °C and examined by CLSM. In the flow cytometry study, cells were incubated with POD, PT-NPs, PTV-NPs, or PCV-NPs (equal to 2 μg/mL of VK3) for 12 h, treated with DCFH- DA at 37 °C for 30 min, followed by flow cytometer detection.

### 2.11. Evaluating intracellular ROS-triggered drug release

Cells were seeded onto a 6-well plate (1 × 10^6^ cells/well) and cultivated overnight. Then, cells were incubated with PT-NPs, PTV-NPs, or PCV-NPs for 8 h, 12 h, 24 h, 36 h or 48 h at a POD-equivalent concentration of 5 μg/mL, respectively. Thereafter, the collected cells were sonicated by an ultrasonicator probe (SCIENTZ-IID) in an ice bath. Methanol/chloroform (1:3, v/v) was used to extract the monomer drugs and collected by centrifugation. To determine the drug concentration, we employed HPLC, as described above.

### 2.12. *In vitro* cytotoxicity assay

*In vitro* anticancer effects of different formulations of drugs were examined by CCK-8 protocol. Cells were seeded into 96-well plates (5 × 10^3^ cells/well) and cultured overnight. Subsequently, cells were incubated with POD, VK3, POD + VK3, PT-NPs, PTV-NPs, or PCV-NPs at various doses for two days. Thereafter, to each well, 10 μL CCK-8 solution was added and incubated for a further 4 h. The absorbance measurement of each well was taken at 490 nm using a microplate reader.

### 2.13. *In vivo* antitumor assay

MCF-7 tumor-bearing mice were used to investigate the antitumor efficacy with tumor size at about 100 mm^3^. In brief, mice were randomly divided into eight groups (six mice in each group). The mice were treated with PBS, POD, VK3, PDO + VK3, PT-NPs, PTV-NPs, or PCV-NPs, respectively, through intravenous tail injection three times equal to POD 10 mg/kg at 0, 3, and 6 days. The weight of mice and tumor volume were recorded every two days. The equation, *V* = L × *S*^2^/2, was employed to calculate the tumor volume, where V, L, and S denote volume, long tumor diameter, and short tumor diameter, respectively. On day 14, the mice were sacrificed, and the tumor tissues were excised and weighted. Moreover, the main organs, including the heart, liver, spleen, lung, kidney, were also collected and embedded in paraffin, sectioned at a thickness of 3 μm, stained with hematoxylin and eosin (H&E), and then examined using an optical microscope for histopathological analysis.

### 2.14. Statistical analysis

Numerical data were presented as mean ± standard deviation (SD). The significance of the difference between more than two groups was analyzed with Student's *t*-test. *P*-values less than .05 were considered significant.

## 3. Results and discussion

### 3.1. Synthesis and characterization of POD prodrug

**Figure 1.**
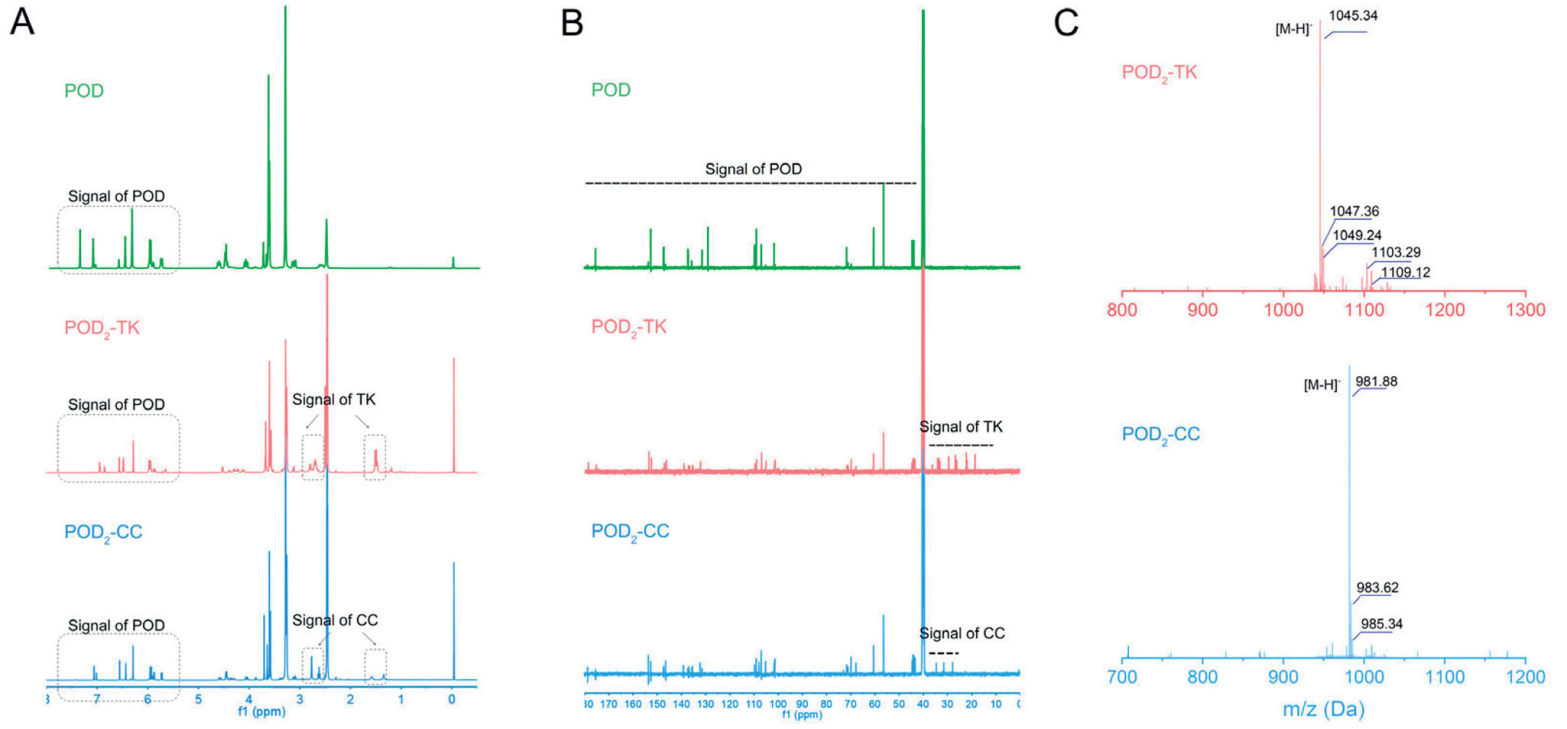
Characterization of prodrugs. (A,B) ^1^H NMR spectrums and ^13 ^C NMR spectrums (B) of POD, POD_2_-TK, and POD_2_-CC in DMSO-*d6*. (C) MS spectrums of POD_2_-TK and POD_2_-CC.

To synthesize ROS-sensitive dimer prodrug and the controlled ROS-insensitive dimer prodrug (denoted as POD_2_-TK and POD_2_-CC, respectively), two POD monomers were linked via a ROS-sensitive TK or ROS-insensitive azelaic acid. The synthesis route of TK, POD_2_-TK, and POD_2_-CC are displayed in Scheme S1. Firstly, the TK linker was prepared as described previously (Hu et al., 2017), and verified by ^1^H NMR and mass spectrometer (Figure S1 and S2). To obtain POD_2_-TK, two POD monomers were subjected to an esterification reaction with the carboxyl group of TK linker. The ^1^H NMR spectrum of POD_2_-TK (Figure 1(A)) showed the chemical shift of TK in the 1.5, 2.7, and 2.8 ppm, and the chemical shift of benzene ring on POD at 6.5–7.4 ppm. Similarly, in the ^13 ^C NMR spectrums, the chemical shifts of POD were presented in 43–175 ppm, while the chemical shifts of TK were presented in 20–40 ppm. These results confirmed that the POD_2_-TK was successfully prepared. In support of the successful synthesis of POD_2_-TK was the consistency between the theoretically calculated value and the peak value in an MS (Figure 1(B)). The ^1^H NMR, ^13 ^C NMR, and MS results also confirmed the successful synthesis of POD_2_-CC (Figure 1(A,B)).

### 3.2. NPs preparation and characterization

**Figure 2.**
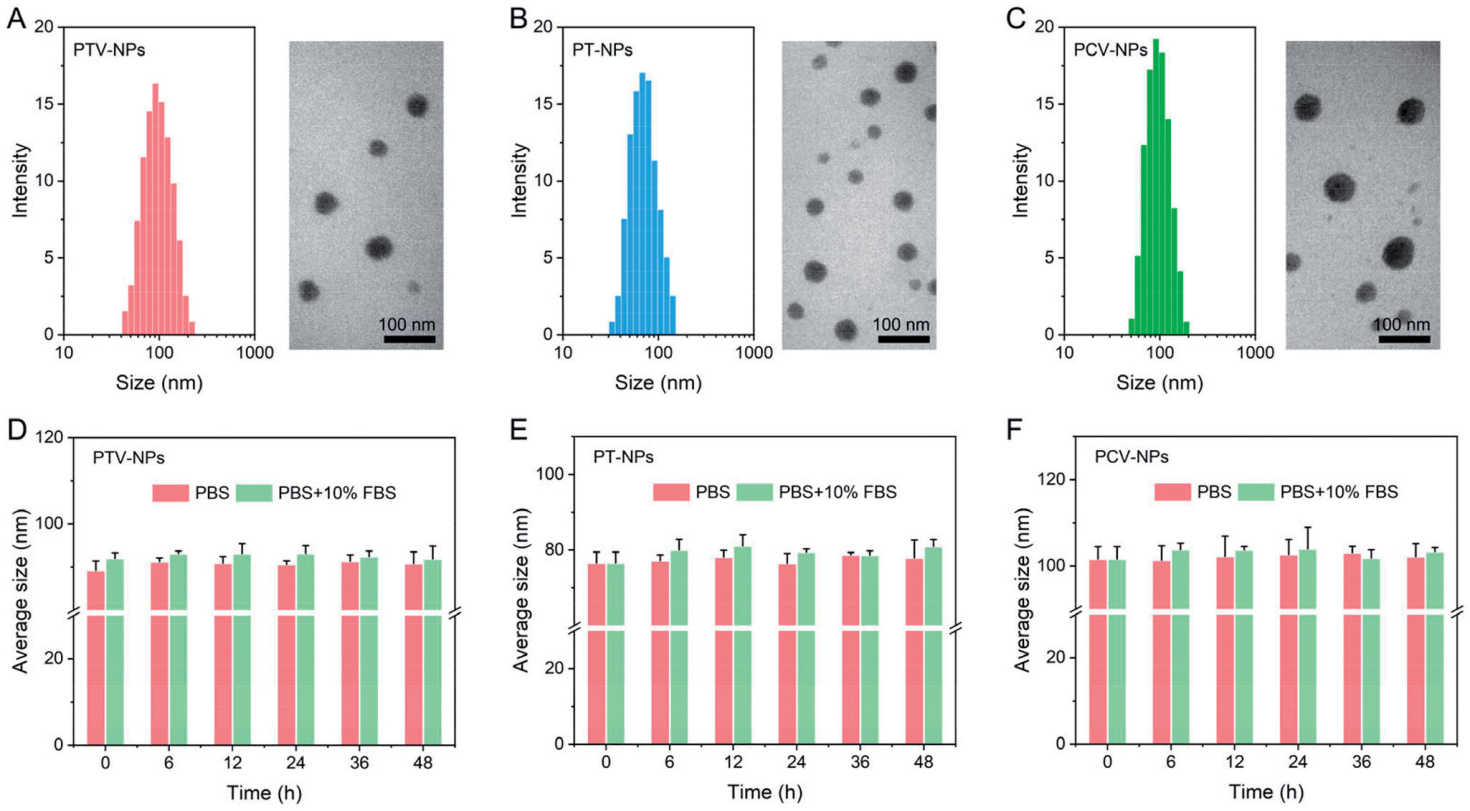
Characterization of NPs. DLS results and TEM images of PTV-NPs (A), PT-NPs (B), and PCV-NPs (C). Size changes of PTV-NPs (D), PT-NPs (E), and PCV-NPs (F) in PBS (pH 7.4) without or with 10% FBS at different incubation times. Data showed as mean ± SD (*n* = 3).

POD dimer-based NPs were prepared by the simple nanoprecipitation method. FDA-approved biocompatible triblock copolymer Pluronic F127 (PEO-PPO-PEO) was added to improve the stability and bioavailability of NPs. To achieve the goal of selective and self-amplification drug release, POD_2_-TK, VK3, and F127 were co-assembled into NPs and denoted as PTV-NPs. For the control, POD_2_-TK and F127 co-assembled NPs, and POD_2_-CC/VK3/F127 co-formed NPs were prepared; these were coded PT-NPs and PCV-NPs, respectively. Of note, all NPs exhibited a high DLC of POD with over 40% (Table S1), remarkably higher than conventionally POD-based delivery systems (usually less than 10, wt%) (Feng et al., 2020). To further confirm the DLC of homodimer prodrug-based NPs was higher than free drug-loaded NPs, the NPs formed by free POD and F127 were also prepared using the same feeding ratio and named as POD-NPs. As shown in Table S1, the DLC and DEE of POD in POD-NPs was (13.7 ± 1.8)% and (58.3 ± 2.9)%, respectively, these results were significantly lower than that of prodrug-based NPs. DLS and TEM characterization allowed for the analysis of the physicochemical properties of NPs. As illustrated in Figure 2(A–C), the three NPs were characterized by a spheroid morphology and uniform particle size (around 70–100 nm) with a narrow distribution (PDI < 0.25). This was suitable for achieving the passive targeting to tumor tissue via the EPR effect (Zhu et al., 2018). The negative charge of PTV-NPs, PT-NPs, and PCV-NPs was mainly because of the hydrogen bonds between the ether bond on the PEG segment and the anions in the PBS solution (Ou et al., 2019). The negative surface charge of NPs can decrease protein adsorption and strengthen its blood circulation (Behzadi et al., 2017). Furthermore, the colloidal stability of these NPs was studied in PBS (pH 7.4) with or without 10% FBS. The particle size of all NPs exhibited minor changes after 48-h incubation (Figure 2(D–F)), demonstrating the good colloidal stability of three NPs.

### 3.3. ROS-responsive evaluation

PTV-NPs were expected to respond to ROS to achieve on-demand drug release. Therefore, we needed to investigate the efficiency of PTV-NPs response to ROS. To verify the ROS-responsiveness of PTV-NPs, three experiments were performed: We monitored the degradation of POD_2_-TK by HPLC, evaluated the disassembled PTV-NPs, and tested the *in vitro* drug release behavior of PTV-NPs.

**Figure 3.**
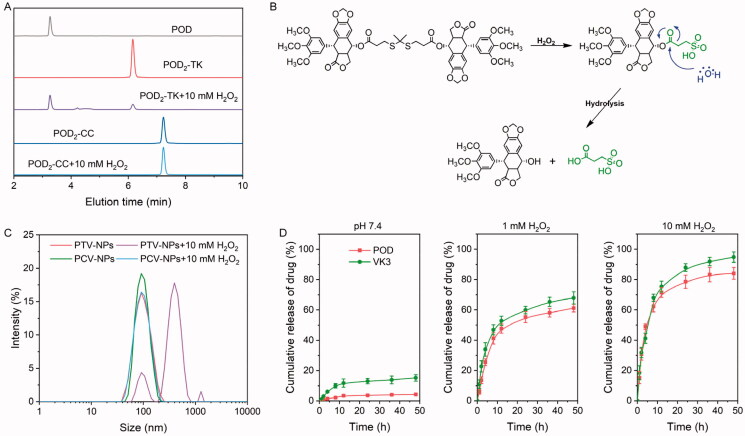
ROS-responsive evaluation of prodrugs and NPs. (A) HPLC profiles of POD_2_-TK and POD_2_-CC under the 10 mM H_2_O_2_ condition. (B) The possible cleavable mechanism of POD_2_-TK triggered by H_2_O_2_. (C) The particle size of PTV-NPs and PCV-NPs after treatment with 10 mM H_2_O_2_. (D) *In vitro* drug release of PTV-NPs at various conditions (data showed as mean ± SD, *n* = 3).

HPLC analysis (Figure 3(A)) showed a monodispersed peak of POD, POD_2_-TK, and POD_2_-CC at an elution time of 5.3 min, 4.3 min, and 6.5 min, respectively. These results demonstrated the high purity of the two prodrugs. Following 8-h incubation of POD_2_-TK with 10.0 mM H_2_O_2_, the peak belonging to POD appeared in the POD_2_-TK spectrum, demonstrating the effective conversion of POD_2_-TK to POD monomer. On the contrary, no peak for POD appeared in the POD_2_-CC spectrum under the same treatment. These results suggest that POD_2_-TK can be degraded successfully to an active POD monomer under a ROS aggregation environment. The underlying mechanism of H_2_O_2_-triggered degradation of POD_2_-TK is outlined in Figure 3(B). The conversation of POD_2_-TK to active POD may occur in the following three steps (Pei et al., 2018): (i) H_2_O_2_-induce TK linker cleaved to two sylfydyl; (ii) the sulfydyl is oxidated to hydrophilic sulfoacid; (iii) prodrug hydrolysis to active POD.

The cleavage of POD_2_-TK could break the stability of NPs and cause their degradation. The size of PTV-NPs exhibited a sharp change from ∼90 nm to ∼400 nm after incubation in 10.0 mM H_2_O_2_ for 12 h (Figure 3(C)). In the control experiment, the size of PCV-NPs exhibited no apparent changes. These results confirmed the ROS-sensitive potential of PTV-NPs.

We also investigated the *in vitro* drug release. The results demonstrated PTV-NPs released a few POD (<5%) and VK3 (<15%) in PBS (pH 7.4) following a 48 h-incubation (Figure 3(D)), suggesting PTV-NPs have high stability in the physiology condition. The stable nanostructure of PTV-NPs could retard the oxidation of TK linkage under blood circulation and storage. Upon culture of NPs in H_2_O_2_ state, abundant POD and VK3 were released from PTV-NPs. However, a slower release of drug behavior was exhibited at lower concentrations (1 mM H_2_O_2_) with 61.1% and 67.8% of POD and VK3 release after 48 h incubation, respectively. Specifically, by increasing H_2_O_2_ concentration to 10 mM, a visible increase in the release of POD and VK3 was detected with a cumulative drug release of 84.1% and 94.8% for POD and VK3, respectively. In the PCV-NPs, because the C-C bond is insensitive to ROS, only a slight of drugs (below 16%) release from PCV-NPs both in the absence (PBS pH 7.4) and in the presence (10 mM H_2_O_2_) ROS conditions (Figure S3). These release behaviors point to the highly responsive nature of PTV-NPs to H_2_O_2_, which is potentially related to the enhanced drug release at the tumor site caused by higher ROS concentrations in tumor cells than that in normal cells (Xia et al., 2020).

### 3.4. Cellular uptake investigation

**Figure 4.**
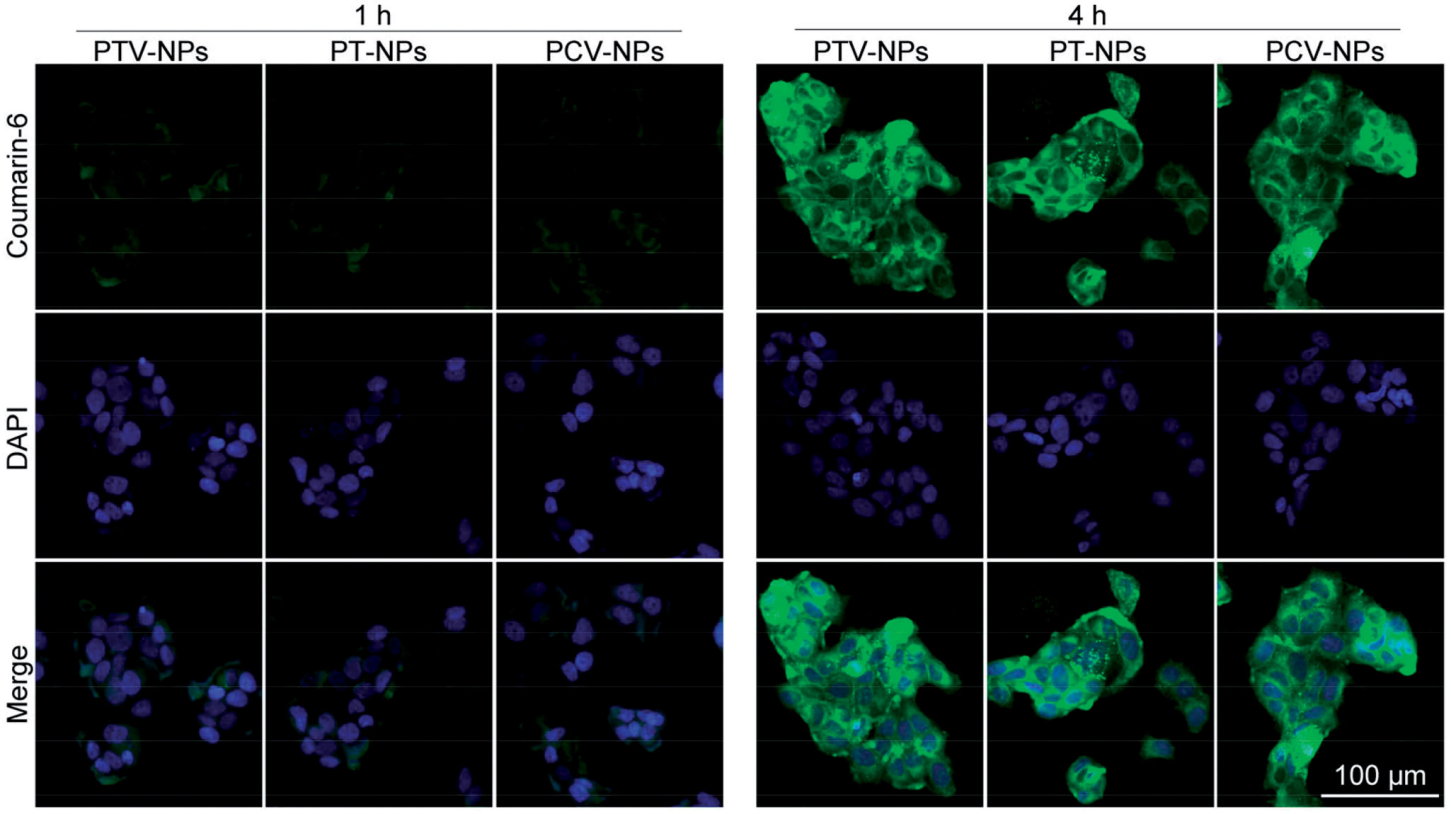
CLSM images of MCF-7 cells after treatment with coumarin-6 loaded PTV-NPs, PT-NPs, or PCV-NPs for 1 and 4 h, respectively.

In this study, the cellular uptake of NPs by MCF-7 cells was assessed by CLSM, with coumarin-6 as the fluorescent probe. Coumarin-6 loaded PT-NPs, PTV-NPs, and PC-NPs were cultured with MCF-7 cells for 2 h or 4 h and examined by CLSM. As shown in Figure 4, weak green fluorescence signals of coumarin-6 were observed in three NPs treatment cells after 1 h incubation, demonstrating that the NPs were internalized successfully by cancer cells. By increasing the treatment duration to 4 h, the green fluorescence intensity of coumarin-6 was more robust in the cytoplasm. These findings suggest that the three NPs can be internalized effectively by cancer cells by a time-dependent increase. The fluorescence intensity in cells treated with the three NPs had no remarkable difference, demonstrating similarity in the internalization process of the three NPs, possibly due to their similar particle sizes and surface properties.

### 3.5. Intracellular ROS production and amplification drug release

Specific ROS generation in cancer cells is another prominent feature of our developed PTV-NPs. To evaluate this capability, cancer cells MCF-7 and normal cells NIH-3T3 were used as the cell models. And the 2′,7-dichlorofluorescindiacetate (DCFH-DA) was employed as a fluorescence probe, which could be rapidly oxidized to dichlorofluorescein (DCF) with green fluorescence by the intracellular ROS (Hu et al., 2017). Because VK3 is the key player in the self-amplification drug release strategy-based drug delivery system, we first evaluated the intracellular ROS amplification ability of VK3. The results show that VK3 could significantly enhance the mean DCF fluorescence intensity in MCF-7 cells in a dose- and time-dependent manner, whereas the mean fluorescence intensity of DCF in NIH-3T3 cells remained unchanged under the same treatment (Figure S4A and S4B). These data demonstrate that VK3 induces ROS augmentation in cancer cells effectively and selectively, consistent with previous reports (Ren et al., 2019; Xia et al., 2020; Tian et al., 2021). Moreover, the ROS production capability of VK3 in MCF-7 cells was impeded by adding the NQO1 inhibitor, dicoumarol (DIC) (Figure S4C). This result suggests that the ROS generation of VK3 in tumor cells is dependent on NQO1.

**Figure 5.**
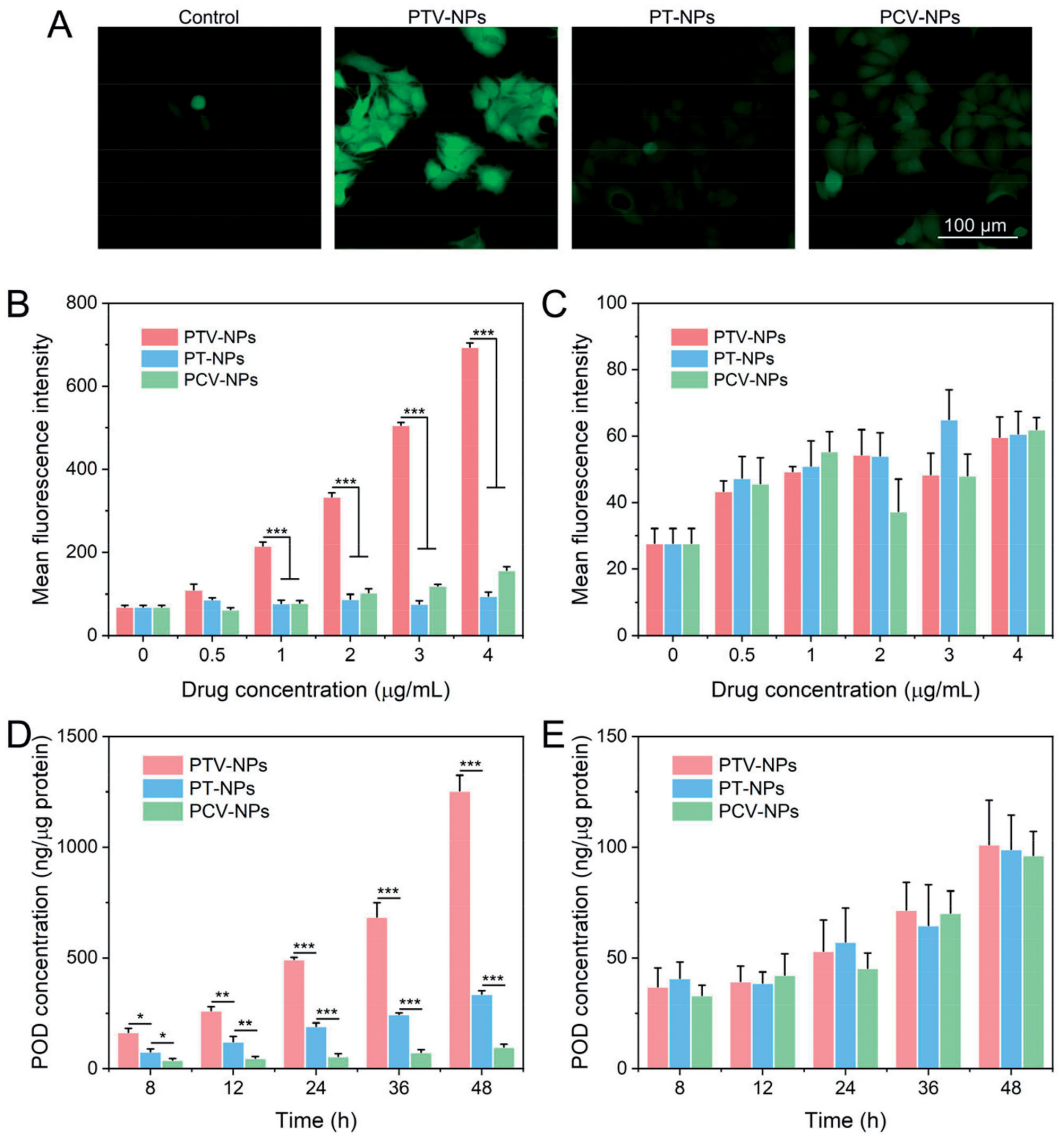
Intracellular ROS amplification and acceleration drug release assay. (A) CLSM images of MCF-7 cells after incubated with PTV-NPs, PT-NPs, and PCV-NPs. (B,C) Flow cytometry quantitative analysis of ROS levels in MCF-7 cells (B) and NIH-3T3 cells (C) incubated with PTV-NPs, PT-NPs, and PCV-NPs at various doses for 12 h. (D,E) HPLC quantitative detection of POD concentration in MCF-7 (D) and NIH-3T3 cells (E) treated with PTV-NPs, PT-NPs, and PCV-NPs at 5 μg/mL (equal to POD) for different times. All the data are exhibited as mean ± SD (*n* = 3); **p < .01, ***p < .001.

Encouraged by the powerful ROS-augmentation capability of VK3, we went ahead to evaluate the intracellular ROS generation capability of prodrug NPs by CLSM and Flow cytometry. Compared with the control group, PTV-NPs could effectively induce ROS production in MCF-7 cells in a dose-dependent manner (Figure 5(A,B)). Moreover, the treatment of PTV-NPs contributed to the highest ROS level at the same concentration. For instance, the mean fluorescence intensity in the PTV-NPs group was 7.3- and 4.4-fold higher than that of the PT-NPs and PCV-NPs group, respectively, when cells were treated with 4 μg/mL drugs. On the contrary, PT-NPs had a negligible effect on the intracellular ROS level in MCF-7 cells, suggesting less efficient drug release. Additionally, the ROS level in MCF-7 cells treated with PCV-NPs was slightly increased, primarily attributed to the passive diffusion of VK3 (16.2%, Figure S3B). More importantly, the ROS concentration in NIH-3T3 cells was not markedly influenced following treatment with these three NPs (Figure 5(c)). These data support the view that PTV-NPs could effectively and specifically amplify ROS levels in tumor cells.

In our hypothesis, VK3 mediated ROS augmentation could, in turn, accelerate the drug release of PTV-NPs. We measured the amount of POD released in MCF-7 cells and NIH-3T3 cells after treatment with prodrug NPs for various periods to demonstrate this concept. The results showed that the MCF-7 cells, the monomer POD in the PTV-NPs treatment group was highest, 2.2-/4.2-, 2.1-/5.6-, 2.6-/8.9-, 3.2-/9.4-, and 4.1-/12.9-fold higher than that of PT-NPs and PCV-NPs treatment group at 1, 2, 4, 8, and 12 h, respectively. Moreover, the active POD concentration in NIH-3T3 cells has no significant difference between the three NPs at the same incubation time. These results could be explained as follows: for PCV-NPs, POD was conjugated with insensitive-linker; thereby, prodrugs hardly convert to monomer drugs, which effectuated the release of a few drugs. For PT-NPs, the abundance of ROS in cancer cells could trigger a part of drug release but insufficiently trigger the release of all drugs. Contrarily, for PTV-NPs, the loaded POD and VK3 would be released from NPs in response to intracellular ROS. The released VK3 produced massive ROS via NQO1 catalysis, which promoted the drug release and contributed to the highest active POD. In normal cells, the absence of ROS and NQO1 resulted in minor drug release from the three NPs. These data demonstrate that the PTV-NPs can induce selective and self-amplification release cargo in tumor cells, this is particularly relevant for tumor-targeted drug delivery and in reducing the side effect of POD.

### 3.6. Cancer-specific cytotoxicity

**Figure 6.**
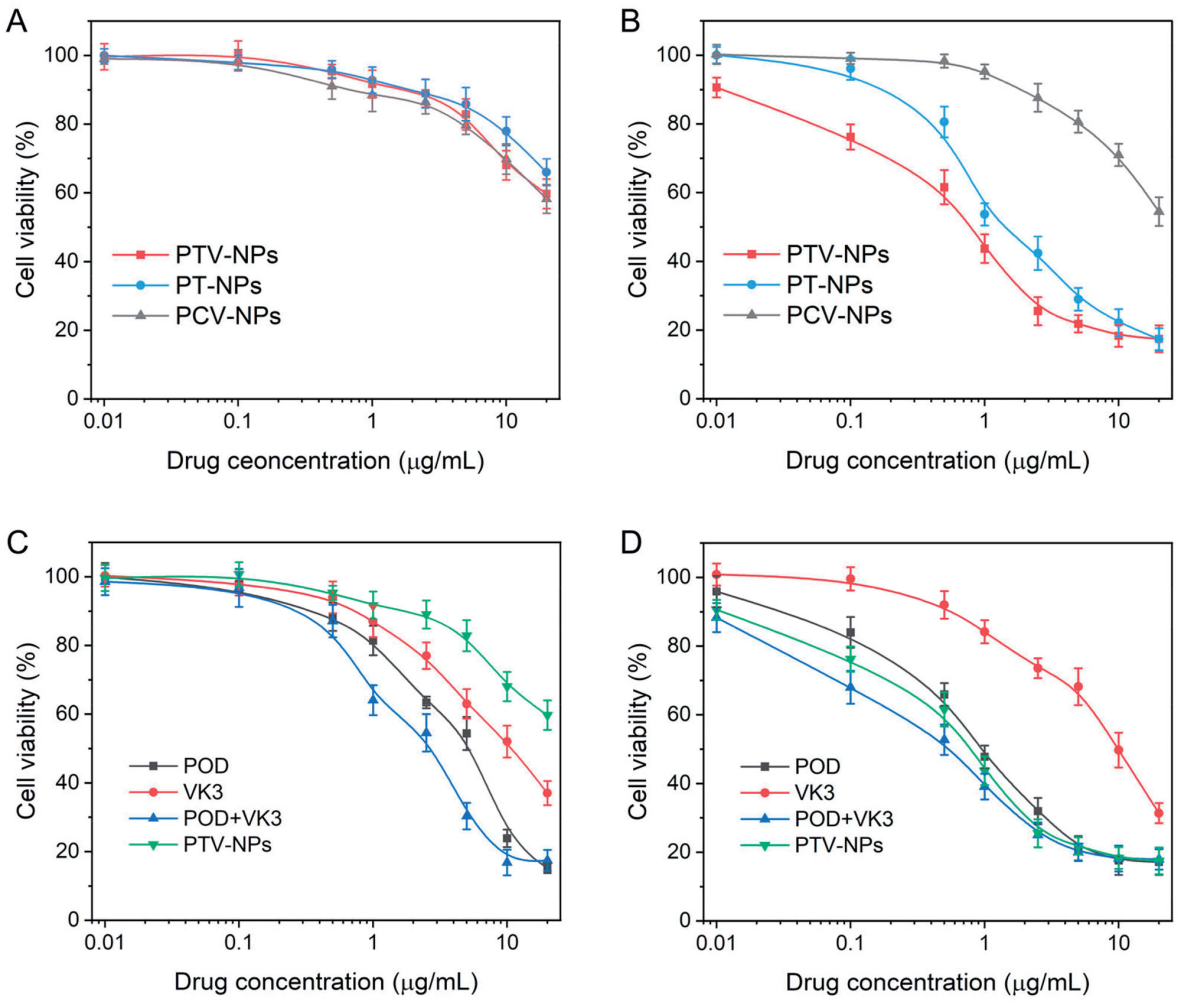
*In vitro* cytotoxicity of different drug formulations. (A–C) Cell viability of NIH-3T3 cells after treatment with NPs (A) or free drugs (C) for 48 h. (B–D) Cell viability of MCF-7 cells after incubation with NPs (B) or free drugs (D) for 48 h. Their results were presented as mean ± SD (*n* = 6).

To validate the benefits of the ROS-mediated self-amplifying drug release delivery system, the cytotoxicity of NPs and free drugs was measured in MCF-7 and NIH-3T3 cells, respectively, using the MTT method. The viability of NIH-3T3 cells had no remarkable difference following treatment with PTV-NPs and PT-NPs. Over 65% of the cells survived at drug doses as high as 20 μg/mL (Figure 6(A)). This can be ascribed to the nonsufficient ROS generation in the normal NIH-3T3 cells, resulting in few drug releases from NPs, insufficient to kill cancer cells. In comparison, both PTV-NPs and PT-NPs showed apparent cytotoxicity against MCF-7 cells (Figure 6(B)). The TK linkage in NPs could be cleaved after internalization by cancer cells with a relatively high ROS concentration, inducing a massive POD release. As expected, the cell toxicity of PTV-NPs and PT-NPs in MCF-7 cells significantly differ. For instance, after treating both NPs with equal to 2.5 μg/mL POD for 48 h, the cell viability in the PTV-NPs group was lower (about 25%), whereas the cell viability in the PT-NPs group was much higher (42%). The underlying mechanism may be that after the inherent ROS triggering, the released VK3 induces ROS production, utilized as a new stimulus to induce much more POD release during the treatment period. This finding was consistent with intracellular drug release quantitative results (Figure 5(D)). Moreover, the IC_50_ value of PTV-NPs against MCF-7 cells was 0.6 μg/mL (POD concentration), while the IC50 value of PTV-NPs against NIH-3T3 cells was over 100 μg/mL (POD concentration) (Table S2). Significantly, the IC_50_ value of PTV-NPs against MCF-7 cells was lower than that of PT-NPs. For the control experiment, the PCT-NPs demonstrated slight cytotoxicity against either MCF-7 cells or NIH-3T3 cells (Figure 6(A,B)). To explain this observation, the ROS-insensitive linker in the PCT-NPs is highly stable under intracellular conditions, resulting in the release of few drugs. Moreover, the IC_50_ of free POD, VK3, and the combination (POD + VK3) was calculated to be 7.1, 13.4, and 2.3 μg/mL for NIH-3T3 cells, respectively, and 0.9, 11.3 and 0.3 μg/mL for MCF-7 cells, respectively (Figure 6(C,D), Table S2). The anti-proliferative activity of free drugs had no obvious between MCF-7 and NIH3T3 cells, suggesting the free POD has severe side effects. These data demonstrate that the PTV-NPs could be targeting deliver the drug to ROS-rich tumor cells, effectively inducing tumor-specific cytotoxicity and minimize the undesired side effects *in vitro*.

### 3.8. *In vivo* antitumor studies

**Figure 7.**
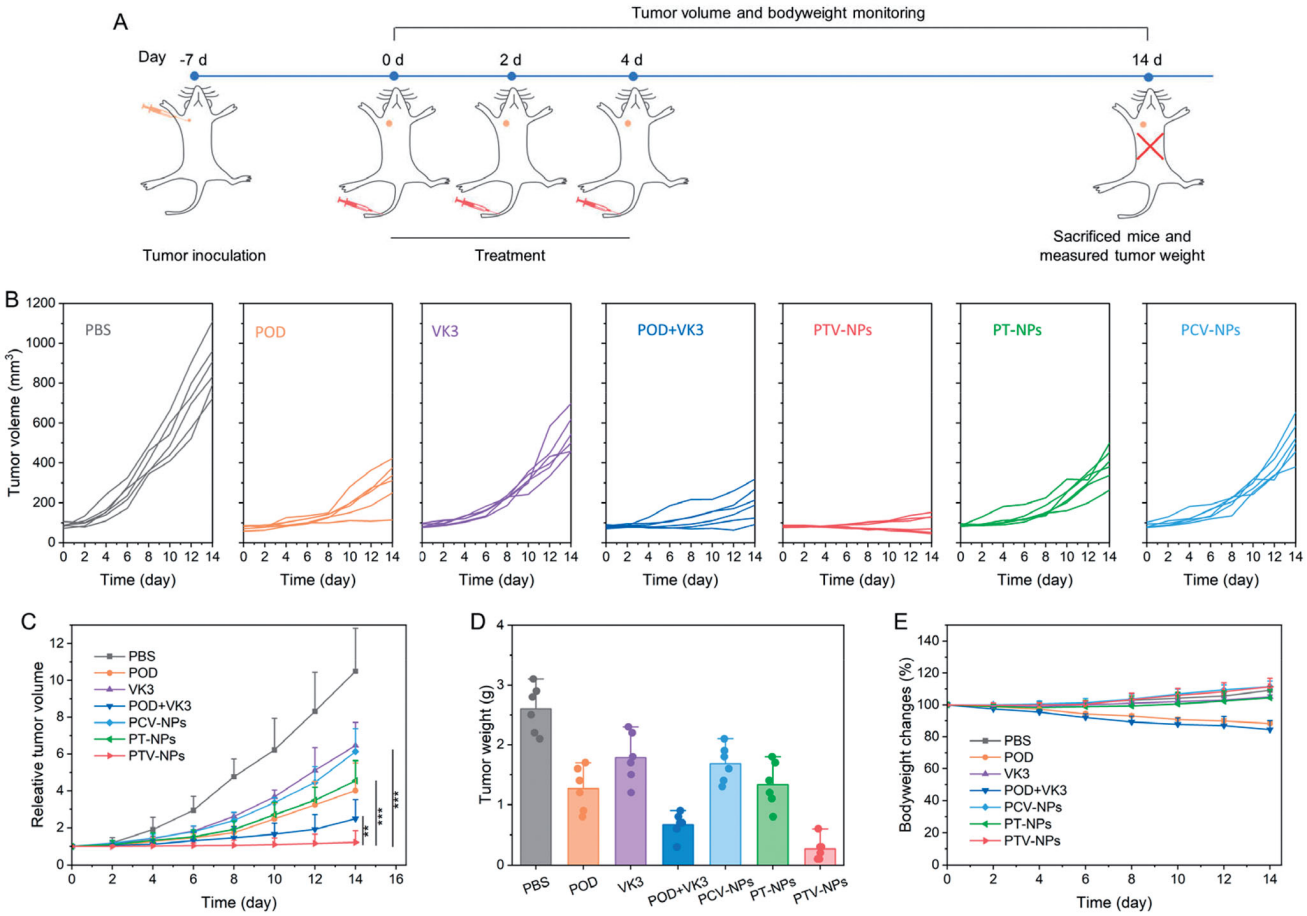
*In vivo* antitumor investigation. (A) Treatment schedule for PTV-NPs against MCF-7 tumor-bearing mice. (B–D) Tumor growth curves of each mouse (B), relative tumor growth curves (C), and tumor weight on day 14 (D) in the different groups after treatment with PBS, POD, VK3, POD + VK3, PTV-NPs, PT-NPs, or PCV-NPs. (E) Mice body weight changes during the therapy. Data are displayed as mean ± SD (*n* = 6).

Inspired by the excellent antitumor efficiency of PTV-NPs *in vitro*, we proceeded to evaluate *in vivo* tumor therapeutic efficacy of PTV-NPs in MCF-7 tumor-bearing mice. At about tumor volume of 80 mm^3^, mice were injected with free drug or NPs *via* the tail vein three times at 15 mg/kg (equal to POD), whereas an equivalent volume of PBS acted as the control group. We monitored the tumor volume during the treatment period (Figure 7(A)). The results showed that the tumors were growing rapidly in PBS groups, and their volumes were increased to 12.3-fold at day 14 compared with day 0 (Figure 7(B,C)), suggesting MCF-7 tumors are highly aggressive. The tumor volumes also increased to 13.2- and 5.6-fold after treatment with POD and POD + VK3, respectively. Low bioavailability could explain the insufficient therapeutic efficacy of free drugs. As expected, the tumor growth in the PTV-NPs treatment group was noticeably obstructed. PT-NPs also exerted a medium antitumor effect, owing to intracellular ROS-triggered partial release of POD. Moreover, PCV-NPs exhibited the worst tumor inhibition effect scribe to insufficient drug release. Tumor weight at the end of the study echoed the excellent antitumor effect of PTV-NPs, which was significantly lower than other groups (Figure 7(D)). Additionally, the remarked decrease in body weight of the POD and POD + VK3 treatment group suggested the toxicity of the free drug (Figure 7(E)). On the contrary, we found no noticeable bodyweight variation in the PTV-NPs treatment group, confirming its upstanding bioavailability and nontoxicity. Simultaneously, histological analysis shows that no obvious pathological abnormalities are observed in the heart, liver, spleen, lung, and kidney during the treatment (Figure S5). These results demonstrate that efficient ROS production and self-amplification drug contributes to excellent antitumor efficacy with no systemic side effects of PTV-NPs.

## 4. Conclusion

In summary, a novel ROS-sensitive POD dimeric prodrug was developed in this study by incorporating VK3 and F127 into NPs. PTV-NPs can selectively increase the ROS level in cancer cells *in vitro* and *in vivo*. Notably, the consequently generated ROS significantly promotes the POD dimeric prodrug conversion to active POD. This strategy can remarkably decrease the side effect of anticancer drugs and increase the antitumor efficacy. This simple design realizes high drug loading and rapid and complete drug release and offers a self-amplification drug release system as a new therapeutic strategy for developing DPNS with increased tumor specificity.

## Supplementary Material

Supplemental Material
