# Supplementary material for: ROS-Activated homodimeric podophyllotoxin nanomedicine with self-accelerating drug release for efficient cancer eradication
Source: Drug Deliv. 2021 Nov 8;28(1):2361–72. doi: 10.1080/10717544.2021.1995076 (PMC8583822; doi:10.1080/10717544.2021.1995076)
Supplement: Supplemental Material [file IDRD_A_1995076_SM4059.docx]

**ROS-Activated Homodimeric Podophyllotoxin Nanomedicine with Self-accelerating Drug Release for Efficient Cancer Eradication**

Bingfeng Liang^1,2^, Dangxia Zhou^1*^

1. Department of Pathology, School of Basic Medicine, Xi’an Jiaotong University Health Science Center, Xi’an, China, 710061

2. Department of Nursing, Hebei Women's Vocational College, Shijiazhuang, China, 050091

***Corresponding author:**

Dangxia Zhou

**Address:** Department of Pathology, School of Basic Medicine, Xi’an Jiaotong University Health Science Center, Xi’an, China, 710061

**Email:** zhoudxdr@163.com

**Supporting Figures**





**Scheme S1.** The synthesis routes of TK (A), POD_2_-TK (B), and POD_2_-CC (D).


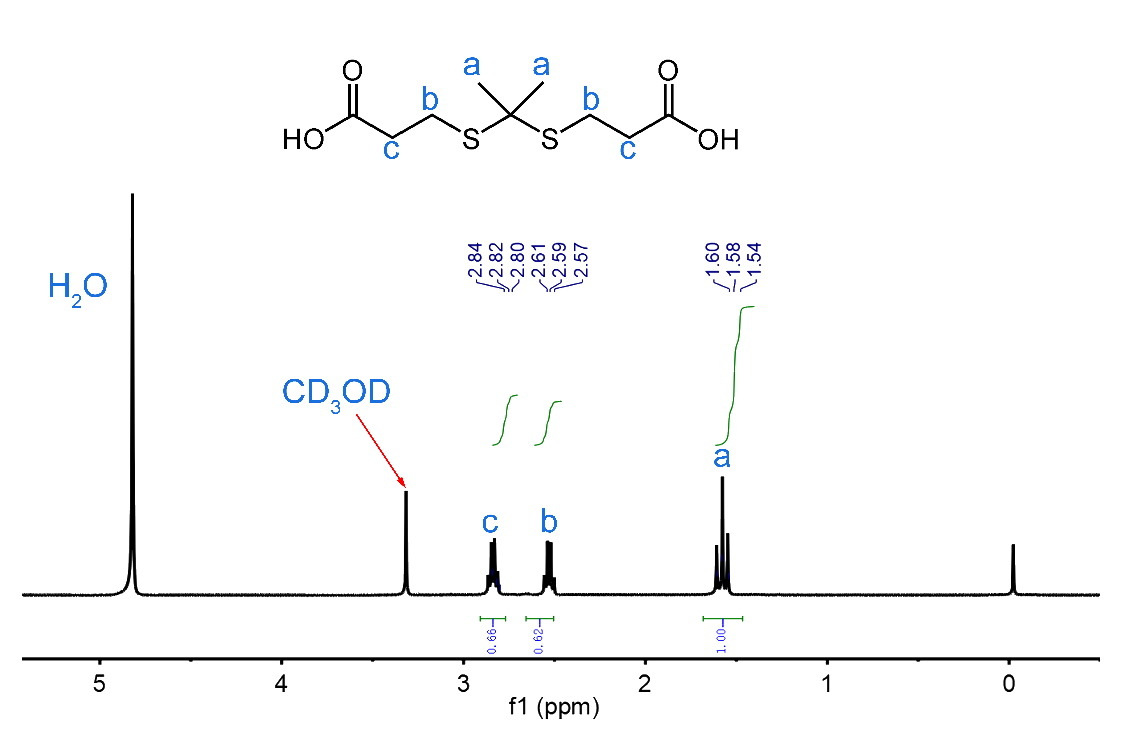


**Fig. S1** ^1^H NMR spectrum of TK in CD_3_OD.


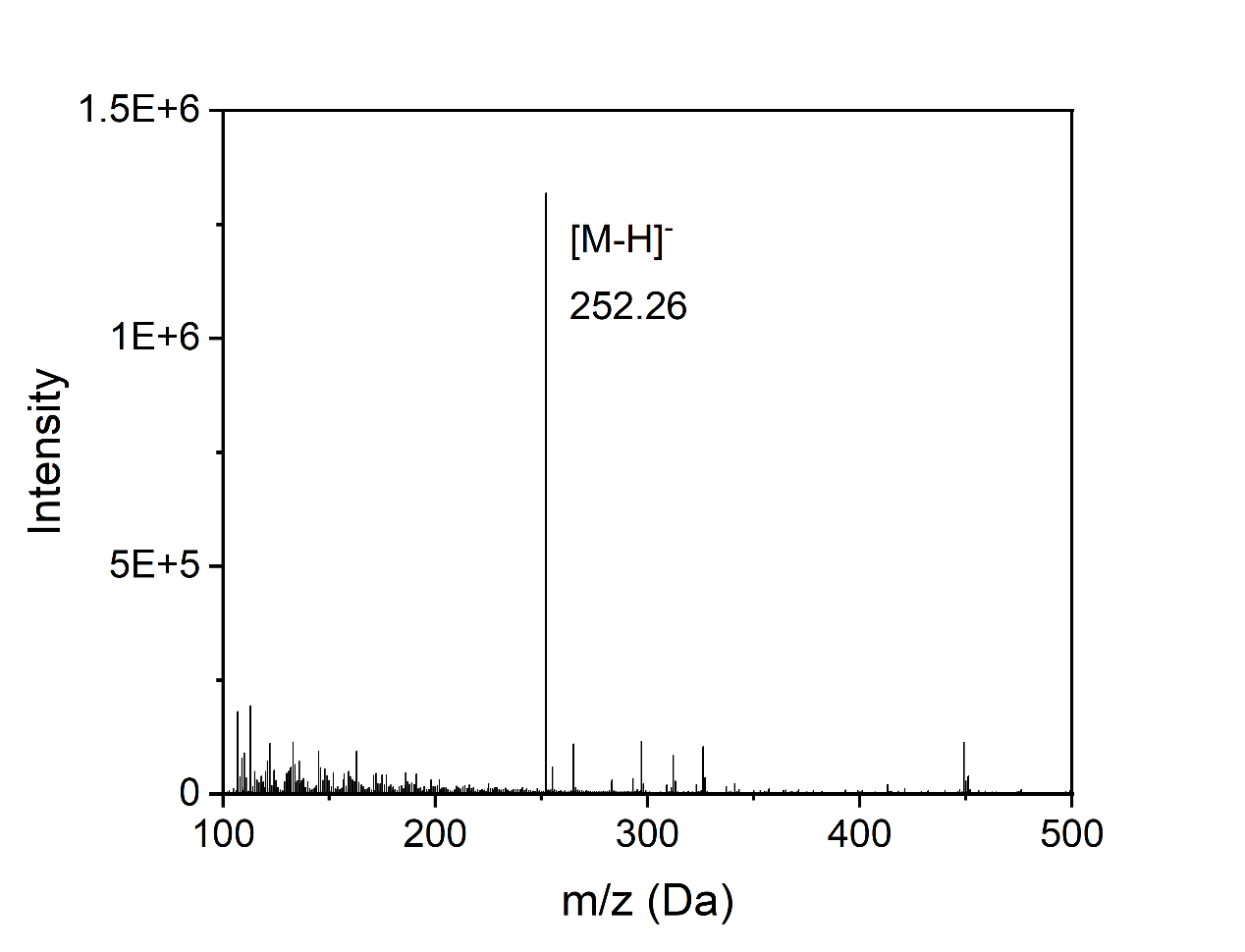


**Fig. S2** Mass spectrum of TK.


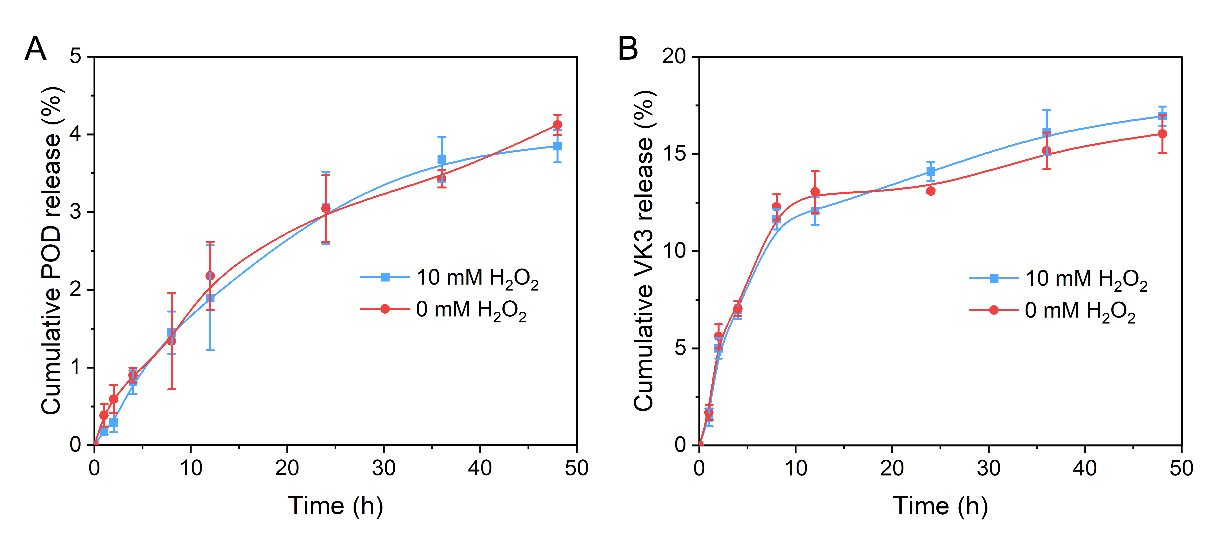


**Fig. S3** *In vitro* release POD (A) and VK3 (B) form PCV-NPs after cultured in PBS (7.4) with or without 10 mM H_2_O_2_.


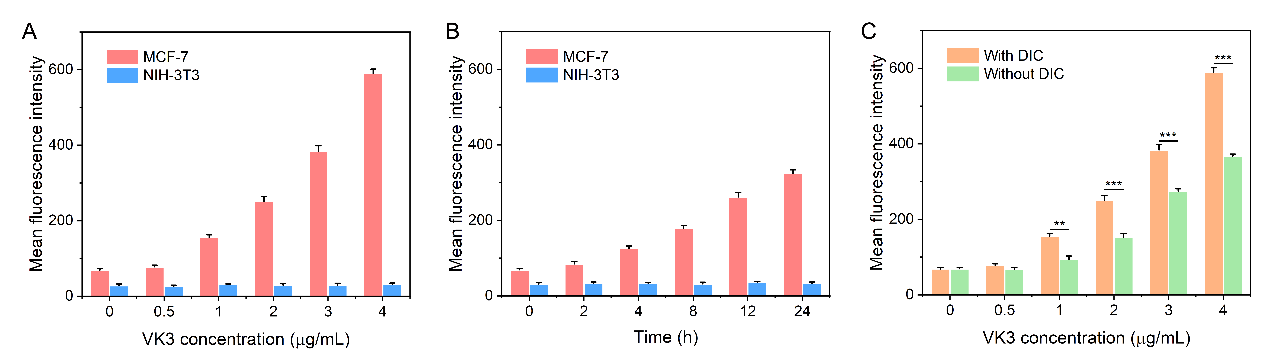


**Fig. S4** Intracellular VK3 induced ROS generation study. A-B: Mean fluorescence intensity in MCF-7 and NIH-3T3 cells after treated with VK3 at different concentration for 12 h (A), or incubated with 2 μg/mL VK3 for different times (B). C: Mean fluorescence intensity in MCF-7 cells after treated with VK3 at different dose for 12 h with or without DIC pretreated. All data were showed as mean ± SD, *n* = 3, ** *p* < 0.01, *** *p* < 0.001.


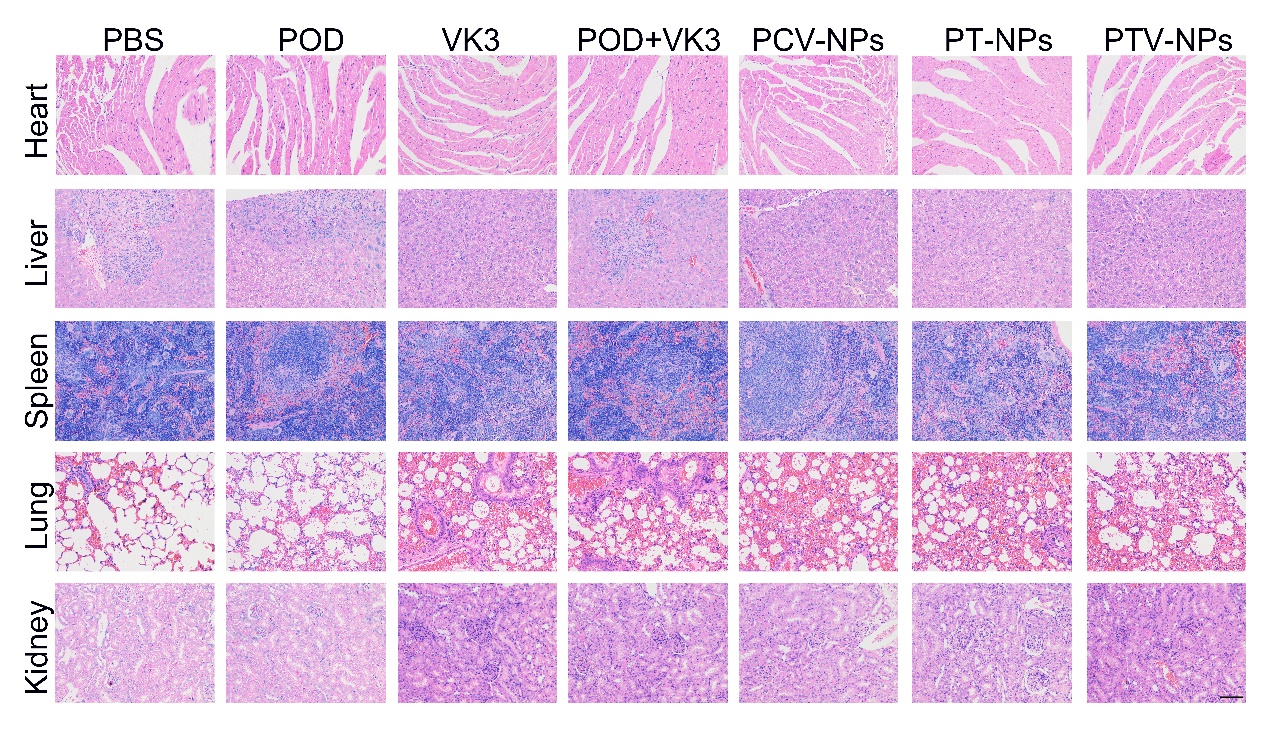


**Fig. S5** H&E staining on the tumor sections of the mice. The organs were collected at day 14 post treatment. Scale bar represents 25 μm.

**Supporting Tables**

| **Table S1.** Characterization of NPs. Data are represented with mean ± SD (*n* = 3). | | | | | | | |
| --- | --- | --- | --- | --- | --- | --- | --- |
| NPs | Size  (nm) | PDI | Zeta potential  (mV) | DLC (100%) | | DEE (%) | |
|  |  |  |  | POD | VK3 | POD | VK3 |
| PTV-NPs | 89.2 ± 2.2 | 0.224 ± 0.020 | -(31.0 ± 1.5) | 43.5 ± 2.3 | 3.4 ± 0.7 | 75.2 ± 3.1 | 63.5 ± 2.3 |
| PCV-NPs | 101.7 ± 2.9 | 0.184 ± 0.011 | -(-33.8 ± 0.7) | 42.3 ± 2.2 | 3.7 ± 0.4 | 67.5 ± 1.5 | 68.4 ± 1.6 |
| PT-NPs | 76.5 ± 2.9 | 0.202 ± 0.013 | -(33.6 ± 1.9) | 47.4 ± 1.4 | - | 81.6 ± 3.2 | - |
| POD-NPs | 187.4 ± 1.5 | 0.432 ± 0.174 | -(28.7 ± 2.9) | 13.7 ± 1.8 | - | 58.3 ± 2.9 | - |

| **Table S2.** IC_50_ values of difference drug formulations against MCF-7 and NIH-3T3 cells (μg/mL) for 48 h. | | | | | | |
| --- | --- | --- | --- | --- | --- | --- |
| Cells | POD | VK3 | POD+VK3 | PTV-NPs | PT-NPs | PCV-NPs |
| MCF-7 | 0.9 | 11.3 | 0.3 | 0.6 | 8.5 | >100 |
| NIH-3T3 | 7.1 | 13.4 | 2.3 | >100 | >100 | >100 |
| The IC_50_ value was calculated by GraphPad Prism 6 software. | | | | | | |
